# Diversity of the Bacterial Microbiome Associated With the Endosphere and Rhizosphere of Different Cassava (*Manihot esculenta* Crantz) Genotypes

**DOI:** 10.3389/fmicb.2021.729022

**Published:** 2021-09-30

**Authors:** Jingwen Ha, Yu Gao, Rui Zhang, Ke Li, Yijie Zhang, Xiaolei Niu, Xin Chen, Kai Luo, Yinhua Chen

**Affiliations:** ^1^Hainan Key Laboratory for the Sustainable Utilization of Tropical Bioresources, Hainan University, Haikou, China; ^2^Institute of Tropical Bioscience and Biotechnology, Chinese Academy of Tropical Agricultural Sciences, Haikou, China

**Keywords:** bacterial diversity, cassava, endosphere, rhizosphere, 16S rDNA, metabolites

## Abstract

Root-associated microbial communities play important roles in plant growth and development. However, little attention has been paid to the microbial community structures associated with cassava, which is a staple food for approximately 800 million people worldwide. Here, we studied the diversity and structure of tuber endosphere and rhizosphere bacterial communities in fourteen cassava genotypes: SC5, SC8, SC9, SC205, KU50, R72, XL1, FX01, SC16, 4612, 587, 045, S0061, and 1110. The results of bacterial 16S rDNA sequencing showed that the richness and diversity of bacteria in the rhizosphere were higher than those in the tuber endosphere across the 14 cassava genotypes. After sequencing, 21 phyla and 310 genera were identified in the tuberous roots, and 36 phyla and 906 genera were identified in the rhizosphere soils. The dominant phylum across all tuber samples was *Firmicutes*, and the dominant phyla across all rhizosphere samples were *Actinobacteria*, *Proteobacteria*, and *Acidobacteria*. The numbers of core bacterial taxa within the tuber endospheres and the rhizospheres of all cassava genotypes were 11 and 236, respectively. Principal coordinate analysis and hierarchical cluster analysis demonstrated significant differences in the compositions of rhizosphere soil microbiota associated with the different cassava genotypes. Furthermore, we investigated the metabolic changes in tuber roots of three genotypes, KU50, SC205, and SC9. The result showed that the abundances of *Firmicutes*, *Proteobacteria*, and *Actinobacteria* in tuber samples were positively correlated with organic acids and lipids and negatively correlated with vitamins and cofactors. These results strongly indicate that there are clear differences in the structure and diversity of the bacterial communities associated with different cassava genotypes.

## Introduction

Plants host diverse and abundant microbial communities that can be considered the “second genomes” of plants. Microbial communities that exist in close association with plants can be categorized into three groups: endophytic, epiphytic, and closely associated (Tringe et al., 2005). Plants and their associated microbes interact with each other and form assemblages of genotypes that are often referred to as “holobionts” (Vandenkoornhuyse et al., 2015; Hassani et al., 2018). Plants attract and select for beneficial microbiomes by releasing signal molecules and providing carbon metabolites as root exudates to endosphere and rhizosphere bacteria (Ryu et al., 2004; Guo et al., 2016; Lopes et al., 2016). Plants can influence net ecosystem changes through deposition of secondary metabolites into the rhizosphere that attract or inhibit the growth of specific microorganisms. This rhizodeposition was made up of small-molecular weight metabolites, amino acids, secreted enzymes, mucilage, and cell lysates (Grayston et al., 1998; Paterson and Sim, 2000). Soil microbes utilize this abundant carbon source, thereby implying that selective secretion of specific compounds may encourage beneficial symbiotic and protective relationships whereas secretion of other compounds inhibit pathogenic associations (Hoffland et al., 1992; Holden et al., 1999). A concrete example is the secretion of isoflavones by soybean roots, which attract a mutualist (*Bradyrhizobium japonicum*) and a pathogen (*Phytopthora sojae*) (Morris et al., 1998). In turn, plants benefit from these relationships, as the microbes change key nutrients into more usable forms (Long, 1989; Bolan, 1991; Zhang et al., 2009). The symbioses between plants and the associated microbes play important roles in the development, health and environmental adaptability of the plant hosts (Spor et al., 2011; Berendsen et al., 2012; Yuan et al., 2018). Previous studies on *Arabidopsis thaliana* (Durán et al., 2018), grapevine (Rolli et al., 2015), and citrus (Zhang et al., 2017) have demonstrated that the bacterial community plays an essential role in plant growth through a variety of mechanisms, including increasing nutrient acquisition, promoting plant hormone production, and protecting plants against pathogen attacks (Ritpitakphong et al., 2016; Álvarez-Pérez et al., 2017; Hassani et al., 2018). Many plant-associated microbes can induce systemic resistance in plants (Liu et al., 2019). For example, stem inoculation with the bacterial strains *Bacillus amyloliquefaciens* (GB03) and *Microbacterium imperiale* (MAIIF2a) mitigates *Fusarium* root rot in cassava (Freitas et al., 2019). *Bacillus cereus* AR156 is a plant growth-promoting rhizobacterium (PGPR) that induces resistance against a broad spectrum of pathogens in *A. thaliana* (Niu et al., 2011). Moreover, harnessing the plant microbiome to maximize crop production is increasingly considered a viable and sustainable approach for the future of agriculture (Geddes et al., 2015; Qiu et al., 2019).

Endophytic microbes and rhizosphere exophytic microbes are affected both by their host plants and by environmental stimuli. In some plants, the diversity and composition of endophytic communities are highly variable between cultivars (Liotti et al., 2018; López et al., 2018). For example, two *Rosa* cultivars with different powdery mildew susceptibilities were determined to share only 34.2% of operational taxonomic units (OTUs), and the resistant cultivar had significantly lower fungal diversity than the susceptible cultivar in the early stage of development (Zhao et al., 2018b). Some studies have shown that the species has a stronger influence on bacterial community composition by growing *Populus*, *Quercus*, and *Pinus* in three soils originating from different field sites (Bonito et al., 2014). The host plant species is the most important factor that determine the leaf endophytic bacterial communities collected from 5 species of plants (*Asclepias viridis*, *Ambrosia psilostachya*, *Sorghastrum nutans*, *Panicum virgatum*, and *Ruellia humilis*) (Ding et al., 2013). Moreover, the taxonomic composition of the extraordinarily diverse communities of microorganisms associated with plants is determined partly by the plant genotype (Korkama et al., 2006; Peiffer et al., 2013; van der Heijden and Schlaeppi, 2015). Genotype effects on the fungal and bacterial microbiomes have been detected in *Triticum aestivum* (Simonin et al., 2020), rice (*Oryza sativa*) (Edwards et al., 2015), maize (*Zea mays*) (Walters et al., 2018), and potato (*Solanum tuberosum*) (van Overbeek and van Elsas, 2008; Inceoğlu et al., 2010). The influence of plant genotypes on belowground microbiota can be attributed to differences in plant growth performance, as well as in the varying amounts of nutrients provided to soil through litter and root exudates (Korkama et al., 2006; van der Heijden and Schlaeppi, 2015; Hugoni et al., 2018) and through their symbionts (Smith and Read, 2008; Gorka et al., 2019). Rhizosphere-associated microbes obtain essential nutrients from plants through host root exudates, and crucial nutrients can be converted to more usable forms, including sugars, organic acids, amino acids, and peptides, by microbes before being assimilated by plants (Ryu et al., 2004). Therefore, through the release of a broad variety of secondary metabolites and root exudates, plants have the capacity to drive and shape plant-associated microbial communities (Raaijmakers et al., 2009). The plant genotype, by determining the community structure of its microbial partners, can be expected to exert cascading effects on ecosystem functions related to nutrient cycling. Overall, plant genetic control of the microbial community is of considerable interest for crop plant breeding and for exploring the possibility of designing a “healthy” microbiome (Morella et al., 2020).

Cassava (*Manihot esculenta* Crantz) is a member of the *Euphorbiaceae* family, has strong environmental adaptability and is tolerant of barrenness and drought (Luo, 2005). Owing to its starch-enriched tuberous root, cassava is an important cash crop in tropical and subtropical areas, and it can also be converted into a large number of products; for example, it is a major resource used in the production of starch, biofuel, and animal feed (Utsumi et al., 2012; Okogbenin et al., 2013). Previous studies have shown that the agronomical characteristics of cassava are significantly influenced by different PGPR strains (Suja et al., 2015). Microbial inoculation significantly improved the mineral nutrient uptake, yield, harvest index, and repression of root rot infection in cassava compared with those in uninoculated controls (Hridya et al., 2013). The application of beneficial bacteria plays an important role in increasing plant growth and protecting against pathogen infection in cassava (Freitas et al., 2019). With the development of next-generation sequencing technologies, culture-independent methods have been employed to determine the profiles of the cassava-associated microbial communities by 16S rDNA sequencing. Li et al. (2020) showed that cassava cultivars recruited various endophytic microbial taxa from tuberous roots to affect the ability of root rot resistance. Similarly, structure of microbiomes of cassava genotypes were analyzed, and revealed their potential roles in cassava bacterial blight resistance (Zhang et al., 2021). Such previous studies have been made on cassava-associated bacteria overall, and few have focused on the comprehensive investigation of endophytic and rhizospheric bacteria of cassava genotypes. Besides, we currently have a poor understanding of how tuber metabolites influence microbial community structure.

In this study, fourteen cassava genotypes with significant differences in genetic background were used to investigate the relationship between cassava-associated bacteria and genetic differences among cassava genotypes by 16S rDNA gene tag sequencing analysis. Moreover, we examined the effects of cassava tuber metabolites collected from three typical cassava genotypes on the different bacterial communities from the tuberous roots and rhizosphere soil. Our results will provide new insight into the linkages between cassava-associated bacteria and cassava genotypes.

## Materials and Methods

### Study Sites and Sample Collection

A total of fourteen cassava genotypes (SC5, SC8, SC9, SC205, KU50, R72, XL1, FX01, SC16, 4612, 587, 045, S0061, and 1110) were provided by Prof. Wenquan Wang, and were grown in the same field in Chengmai county, Hainan Province, China (19°85′ N, 110°08′ E, elevation 83 m a.s.l.) (Supplementary Table 1). The average annual temperature at the study location is 23.8°C, the annual precipitation is 1786.1 mm and the average annual sunshine hours are 2,059 h. The soil type at the site is a red loam.

Two samples (tuberous roots and rhizosphere soil) of the fourteen genotypes were taken with three biological replicates in March 2019. The detail of endosphere and rhizosphere microbes sampling was as follows. The whole tuberous roots were taken out and the bulk soil was removed by careful shaking. Soil still adhering to the tubers was collected with sterile tweezers and defined as the rhizosphere soil. In order to remove the majority of rhizosphere-associated microbes and enrich for endophytic microbes, the tubers were washed with water and sterilized, first with 75% alcohol and then with a sodium hypochlorite solution containing 1% active chlorine. Then, the tubers were washed with sterilized water, and cleaned using sterilized filter paper, and placed into sterilized bags. All the samples were stored at −80°C in liquid nitrogen until DNA extraction (Dong et al., 2018). The tuberous roots of three cassava genotypes, KU50, SC205, and SC9, were collected for metabonomic analysis, and three biological replicate samples were taken for each cassava genotype.

### DNA Extraction and Illumina MiSeq Sequencing

The tuber samples were ground into powder by the liquid nitrogen grinding method for the extraction of the endophytic flora. Genomic DNA was extracted from the freeze-dried tuber powder (50 mg) and freeze-dried soil samples (0.20 g) with E.Z.N.A.^TM^ Mag-Bind Soil DNA kits (Omega, United States), following the manufacturer’s instructions. We measured the concentration of the DNA using a Qubit 2.0 (life, United States) to ensure that adequate amounts of high-quality genomic DNA had been extracted. The 16S rDNA V3–V4 amplicon was amplified using KAPA HiFi Hot Start Ready Mix (2×) (TaKaRa Bio Inc., Japan) and individual barcoded primers with gene-specific regions of those primers corresponding to 341F (5′-CCTACGGGNGGCWGCAG-3′) and 805R (5′-GACTACHVGGGTATCTAATCC-3′) (Dethlefsen and Relman, 2011). The polymerase chain reaction amplification conditions were as follows: the reaction mixtures in each tube contained 2 μL of target DNA (10 ng/μL), 15 μL of 2 × KAPA HiFi Hot Start Ready Mix, 1 μL of amplicon PCR forward primer (10 μM), 1 μL of amplicon PCR reverse primer (10 μM), and 11 μL of sterile distilled water, with a total volume of 30 μL. The plate was sealed and polymerase chain reaction (PCR) performed in a thermal instrument (Applied Biosystems 9700, United States) using the following program: the thermal cycling conditions for the primary PCRs consisted of 3 min at 93°C, followed by 5 cycles of 30 s at 94°C, 20 s at 45°C, and 30 s at 65°C, followed by 20 cycles of 20 s at 94°C, 20 s at 55°C, and 30 s at 72°C, and a final extension for 5 min at 72°C. The PCR products were checked using electrophoresis in 1% (w/v) agarose gels in TBE buffer (Tris, boric acid, EDTA) stained with ethidium bromide (EB) and visualized under UV light.

After PCR amplification, quantification of the bacterial 16S rDNA was performed using Qubit3.0 DNA detection kits. Next, the samples were loaded onto an Illumina MiSeq high-throughput sequencing platform for paired-end sequencing (Shao et al., 2017) and sequenced by Sangon BioTech (Shanghai, China). The raw Illumina MiSeq sequences were processed and analyzed using the Quantitative Insights into Microbial Ecology (QIIME) software package (version 1.8.0) (Caporaso et al., 2010). The paired-end reads were merged into longer contigs and quality filtered to remove contigs with lengths < 200 nt, average quality scores of < 20, and contigs containing > 3 nitrogenous bases by PANDAseq. The quality-filtered sequences were subsequently clustered in OTUs at 97% similarity and annotated using the Ribosomal Database Project (RDP) and Silva bacterial databases to determine the phylogeny and relative abundances of the OTUs (Cole et al., 2007). The unclassified OTUs and the reads identified as chimeras through UCHIME (Edgar et al., 2011) were removed from downstream analysis. The raw data were submitted to the NCBI Sequence Read Archive (Accession No. PRJNA750582).

### Extraction of Tuber Metabolites

Twenty-five milligrams (± 2%) of each tuberous sample was accurately weighed out and ground. The sample to be tested was then extracted, and gas chromatography-mass spectrometry (GC-MS) detection was performed. Briefly, GC was performed on an HP-5 MS capillary column (5% phenyl/95% methylpolysiloxane 30 m × 250 μm i.d., 0.25 μm film thickness, Agilent J and W Scientific, Folsom, CA, United States) to separate the derivatives at a constant flow rate of 1 mL/min helium. One microliter of sample was injected in split mode in a 20:1 split ratio by the autosampler. The injection temperature was 280°C, the interface was set to 150°C, and the ion source was adjusted to 230°C. The temperature-increase program was as follows: initial temperature of 60°C for 2 min, 10°C/min increase rate to 300°C and holding at 300°C for 5 min. MS was performed with the full-scan method within a range from 35 to 750 (m/z) (Sangster et al., 2006; Want et al., 2013).

The obtained raw data were converted into the netCDF format (xcms input file format) through Agilent MSD ChemStation (Smith et al., 2006). R (v3.1.3) was used to obtain data matrices, including the mass to charge ratio (m/z), retention time, and peak area (intensity). Metabolite annotations were performed with the AMDIS program. The databases used for annotation were the National Institute of Standards and Technology (NIST) commercial database and the Wiley Registry metabolome database. Among them, the metabolite alkane retention index was used for further qualitative substance analysis according to the retention index provided by the Golm Metabolome Database (GMD),^1^ and most of the substances were further confirmed by standard products.

### Bioinformatics and Statistical Analysis

R software (version 4.0.2) was used for bioinformatics analysis, and certain plots were generated using the “ggplot2” package. The “vegan” package was used to calculate the number of microorganisms and abundance based on the 16S OTU table. For any sample, we used total sum scaling to calculate the relative abundance and expressed the relative abundance as percentages. The richness and diversity statistics including the richness [the abundance-based coverage estimator (ACE)] and the Shannon diversity index were also calculated using mothur (Schloss et al., 2009). The modified pipeline is described on the mothur website. A *t*-test or two-way analysis of variance (ANOVA) with Duncan’s multiple range test was performed for multiple comparisons to determine the significant differences in the total number and α-diversity index of bacteria in the tuberous roots and rhizosphere soil, and Excel 2019 software was used to visualize the differences. All statistical tests performed in this study were considered significant at *P* < 0.05 with SPSS version 20.0 software. Differences were considered significant at *P* < 0.05. The effects of cassava genotypes on the core and unique microbial OTUs in each tuber and the soil environment were analyzed according to the methods provided by Shade and Handelsman (2012) and Zhao et al. (2018a), respectively. The OTUs that consistently appeared in the three biological replicates of all plant genotypes were considered the core microbiome, while the OTUs that were present in all three biological replicates of only one plant genotype were considered unique microbiomes. The significant differences in the microbiome of a given cassava genotype among treatments were tested using one-way ANOVA and the least significant difference (LSD) test (*P* < 0.05). These results were visualized using Venn diagrams. Principal coordinates analyses (PCoAs) based on Bray-Curtis distance were used to evaluate the differences among the microbial communities of the different cassava genotypes in the tuberous roots and rhizosphere soil. Hierarchical clustering analysis (HCA) was performed based on the β-diversity distance matrix, and then the unweighted pair group method with arithmetic mean (UPGMA) algorithm was used to build a cluster tree. Linear discriminant analysis (LDA) and effect size (LEfSe) analyses were performed using the LEfSe tool (Segata et al., 2011). Differences in rhizosphere bacterial abundance were analyzed by LEfSe. The LEfSe analysis used the Kruskal-Wallis rank sum test to detect significantly different abundances and generated LDA scores to estimate the effect size (threshold: ≥ 2).

The relative content (percentage) of each metabolite produced by the three cassava genotypes and their biological roles were determined and then visualized with a stacked column chart to compare the compositions and structures of the tuber metabolites. Unsupervised principal component analysis (PCA) and orthogonal partial least squares discrimination analysis (OPLS-DA) based on the “MetaboAnalyst” package in R were used to compare the compositions and structures of tuber metabolites among the different cassava genotypes and to identify significant differences in tuber metabolites among genotypes. The correlations between metabolites and bacterial phyla were estimated using Mantel tests (type = Spearman) in the “vegan” package. In addition, Pearson correlation analyses were performed with the “corrplot” package and used to reveal correlations between the abundance of the microbial flora and the composition of tuber metabolites.

## Results

### General Characteristics of 16S rDNA Based on Sequencing Data

In this study, we obtained 3,392,789 and 3,648,420 raw reads from the MiSeq sequencing analysis of the two sampling sites (each sampling site consisted of 14 cassava cultivars × 3 biological replicates), with an average of 80,781 and 86,867 reads per sample. After quality filtering, a total of 3,225,194 and 3,418,691 reads were obtained from the two sampling sites, with an average of 76,790 and 81,397 reads obtained in each sample (Supplementary Table 1). The reads were clustered into a total of 3,927 and 239,156 OTUs according to 97% sequence similarity. The taxonomic assignment of the OTUs resulted in the identification of 21 phyla and 310 genera in tuberous roots and 36 phyla and 906 genera in rhizosphere soil (Supplementary Table 2).

### Microbial Taxonomic Analysis at the Phylum Level

**FIGURE 1:**
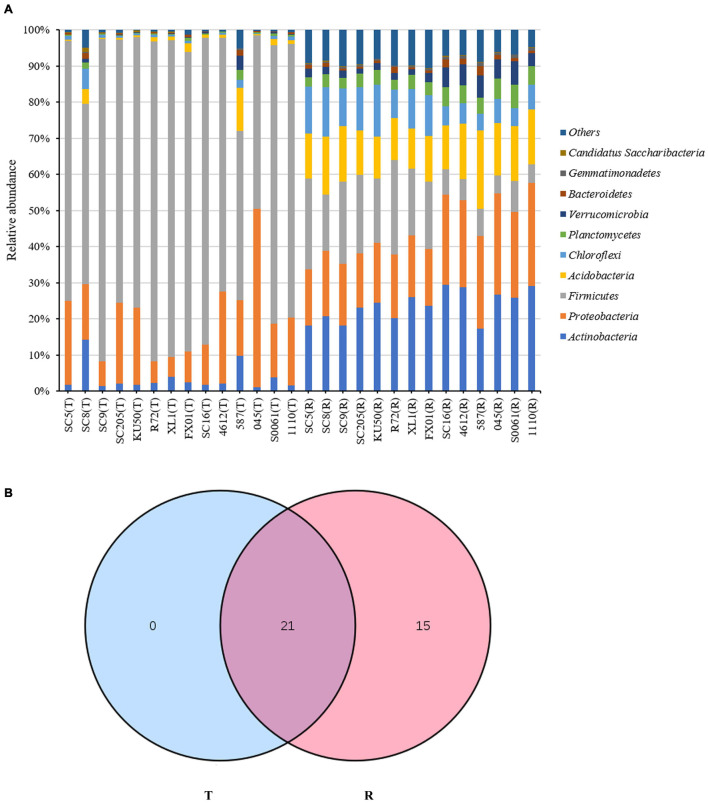
**(A)** Histogram of the relative abundances at the phyla level of the TOP 10 bacterial communities. **(B)** Number of bacterial phyla in tuberous roots and rhizosphere soil of different cassava genotypes. T stands for tuberous roots; R stands for rhizosphere.

The relative abundances of the 10 most abundant phyla (>1% of relative abundance in at least one sample) are shown in Figure 1A and Supplementary Table 2. *Firmicutes* was the dominant phylum (>10% relative abundance) across all tuber samples, accounting for 46.9–89.3% of the total high-quality sequences. *Proteobacteria* and *Actinobacteria* were the next most abundant phyla (>1% relative abundance) in all tuber samples, accounting for 5.6–49.4% and 1.0–14.2% of the total high-quality sequences, respectively. Interestingly, the abundances of *Actinobacteria* in SC8 and *Acidobacteria* in 587 were extremely high compared with those in the other tuber samples. However, among the rhizosphere samples, *Actinobacteria*, *Proteobacteria* and *Acidobacteria* were the dominant phyla (> 10% relative abundance) across all rhizosphere samples, accounting for 17.4–29.5%, 15.2–28.5%, and 11.0–21.8% of the total high-quality sequences, respectively. *Firmicutes*, *Chloroflexi*, *Planctomycetes*, and *Verrucomicrobia* were the next most abundant phyla (> 1% relative abundance) in all rhizosphere samples, accounting for 4.9–26.2%, 4.5–14.4%, 2.7–6.5%, and 1.5–6.5% of the total high-quality sequences, respectively. Among the rhizosphere samples, the abundances of *Firmicutes* and *Acidobacteria* were higher in SC5, SC8, SC9, SC205, KU50, XL1, and FX01 than in the rhizospheres of the other genotypes (Figure 1A). The identities and relative abundances of bacterial phyla between tuberous roots and rhizosphere soil were obviously different. Based on the Venn diagram analysis, 21 phyla in the rhizosphere soil were found to be common to all tuberous samples, and 15 phyla were exclusive to the rhizosphere samples (Figure 1B).

### Comparison of Bacterial Community Structures at the Genus Level

**FIGURE 2:**
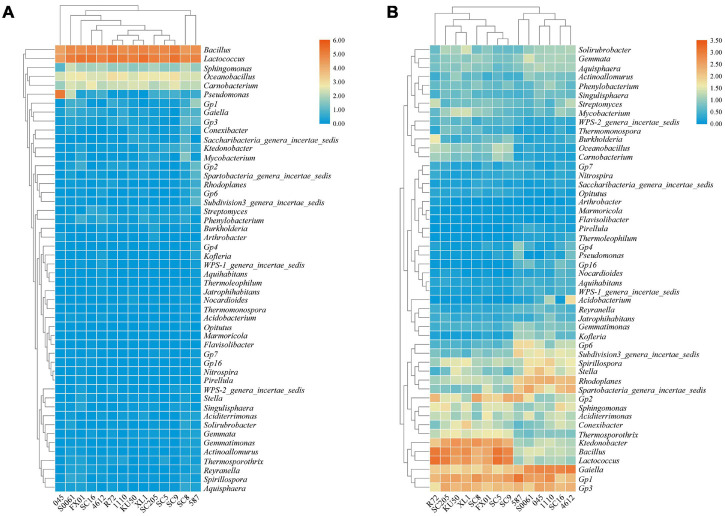
Heatmap of the relative abundances at the genus level of the TOP 50 bacterial communities in **(A)** the tuberous roots and **(B)** the rhizosphere soil of different cassava genotypes.

Based on the heatmap analysis of the relative abundances of the 50 most abundant classified genera, there were clearly significantly different bacterial community structures between the tuberous roots and rhizosphere soil of the fourteen cassava genotypes analyzed (Figure 2). The 50 most abundant genera belonged to 13 phyla (Supplementary Table 3).

**FIGURE 3:**
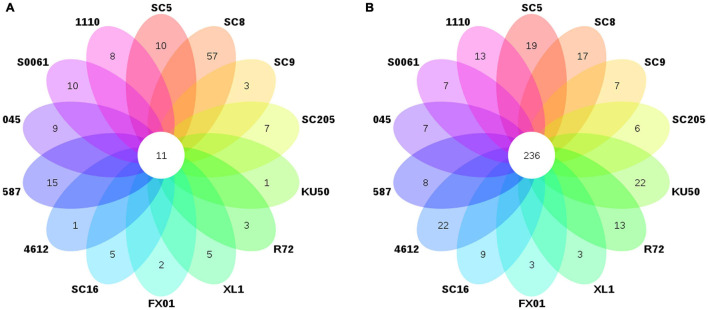
Number of bacterial genera in **(A)** the tuberous roots and **(B)** the rhizosphere soil of different cassava genotypes.

*Lactococcus* and *Bacillus* were the dominant genera (>10% relative abundance) in all tuber samples, accounting for 20.0–41.3% and 18.5–32.7% of the total high-quality sequences, respectively. Interestingly, the abundance of *Pseudomonas* in 045 was much higher than those in the other tuber samples. *Oceanobacillus* and *Carnobacterium* were the next most abundant genera (> 1% relative abundance) in all tuber samples, accounting for 4.1–6.9% and 2.3–5.3% of the total high-quality sequences, respectively (Figure 2A). There were 11 core genera in the tuberous roots, accounting for only 3.5% of the total tuber bacterial community (Figure 3A). The core bacterial genera of the fourteen cassava genotypes included *Lactococcus*, *Bacillus*, *Oceanobacillus*, *Acinetobacter*, *Carnobacterium*, *Sphingomonas*, *Streptococcus*, *Exiguobacterium*, *Leuconostoc*, *Enterococcus*, and *Phenylobacterium*. Among them, 6 genera had significant differences in abundance among genotypes (*P* < 0.05), namely, *Lactococcus*, *Bacillus*, *Oceanobacillus*, *Acinetobacter*, *Streptococcus*, and *Exiguobacterium* (Supplementary Table 4).

The distributions of the genera differed greatly across the different rhizosphere samples. A total of eight genera (*Gp1*, *Gaiella*, *Bacillus*, *Lactococcus*, *Gp3*, *Ktedonobacter*, *Rhodoplanes*, and *Spirillospora*) were highly abundant (>1% relative abundance) in all rhizosphere samples (Figure 2B). The core bacterial genera in the rhizosphere soils remained similar among the different genotypes of cassava. There were 236 core bacterial genera, accounting for 26.0% of the total rhizosphere bacterial community (Figure 3B). The relative abundances of most of the core bacterial genera showed significant differences among genotypes (*P* < 0.05) (Supplementary Table 4).

### Effects of Host Genetics Based on α-Diversity Analysis

**FIGURE 4:**
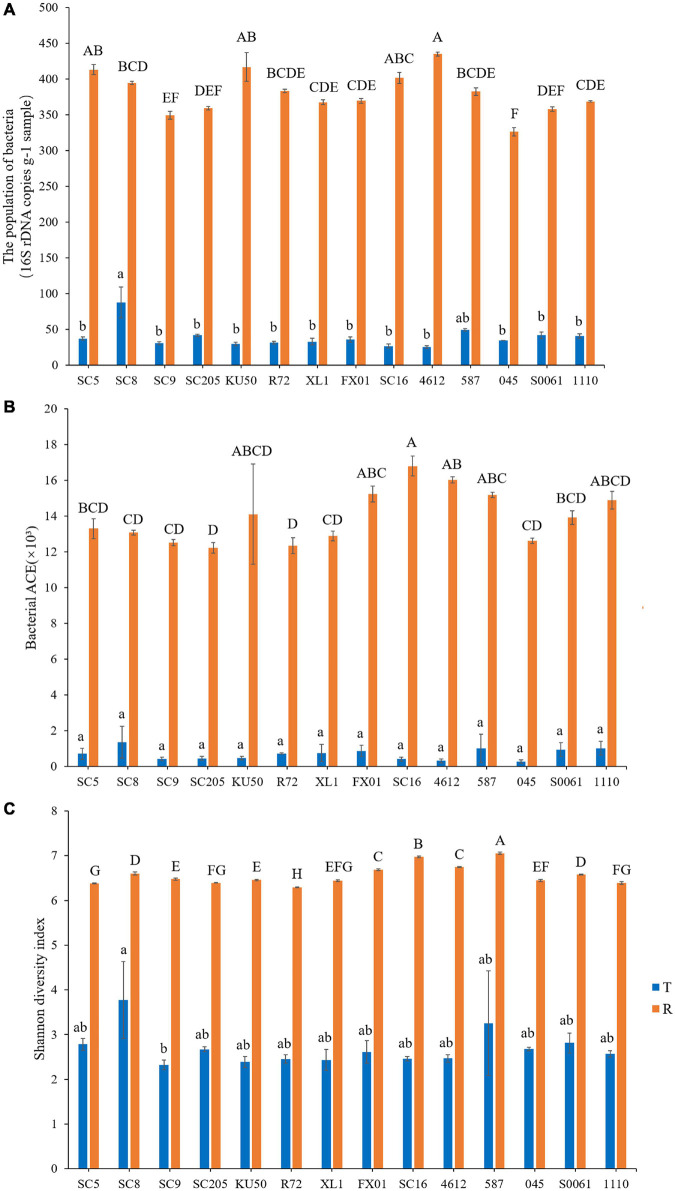
Microbial population **(A)**, richness (abundance-based coverage estimator, ACE) **(B)**, and Shannon diversity **(C)** in the tuberous roots and rhizosphere soil of different cassava genotypes. Error bars represent standard deviations (SDs). Different lowercase letters and capital letters represent significant differences (*P* < 0.05) within tuberous roots and rhizosphere soil according to Duncan test, respectively. T stands for tuberous roots; R stands for rhizosphere.

To compare the α-diversity of samples with different sequence counts, we refined the data (i.e., we randomly picked an equal number of sequences across samples) using QIIME. The rarefaction curves showed the richness of the observed OTUs (Supplementary Figure 1) and indicated that the sequencing depth was sufficient to fully capture the diversity present. Microbial abundance and α-diversity were estimated using the population of bacteria, the bacterial richness (ACE) and the bacterial Shannon index, and had a statistical analysis performed with genotype and sampling site as explanatory variables. The highest richness was detected in the rhizosphere soil samples, which had significantly higher OTU and bacterial numbers and ACE and Shannon index values than the tuber samples. Among the tuber samples, SC8 had the highest number of OTUs (*n* ≥ 306 OTUs), and 4612 had the lowest number of OTUs (*n* = 49 OTUs). Among the rhizosphere soil samples, SC16 had the highest number of OTUs (*n* ≥ 6,858 OTUs), and SC205 had the lowest number of OTUs (*n* ≥ 4,992 OTUs). Further, the differences in the total number and microbial alpha-diversity of bacteria were tested with the *t*-test or two-way analysis of variance (ANOVA) with Duncan’s multiple range test and were considered different at *P* < 0.05. Our results showed that there were no significant differences in the number of bacterial OTUs among the fourteen genotypes (*P* > 0.05) (Supplementary Table 5). Similarly, among the tuber samples, no significant differences in microbial abundance or α-diversity were observed among the fourteen genotypes (*P* > 0.05). Overall, SC8 had the highest number of bacteria and α-diversity (Figure 4 and Supplementary Table 5). However, the comparison of the microbial abundance and α-diversity metrics of the rhizosphere soils revealed disparities in the bacterial number, ACE, and Shannon indices among the fourteen genotypes (*P* = 0.000032, 0.005132, and 8.5974E^–22^, respectively). Furthermore, SC16, 587, 4612, and FX01 showed significantly higher bacterial diversity than the other genotypes (Figure 4 and Supplementary Table 5).

### Effects of Host Genetics Based on β-Diversity Analysis

**FIGURE 5:**
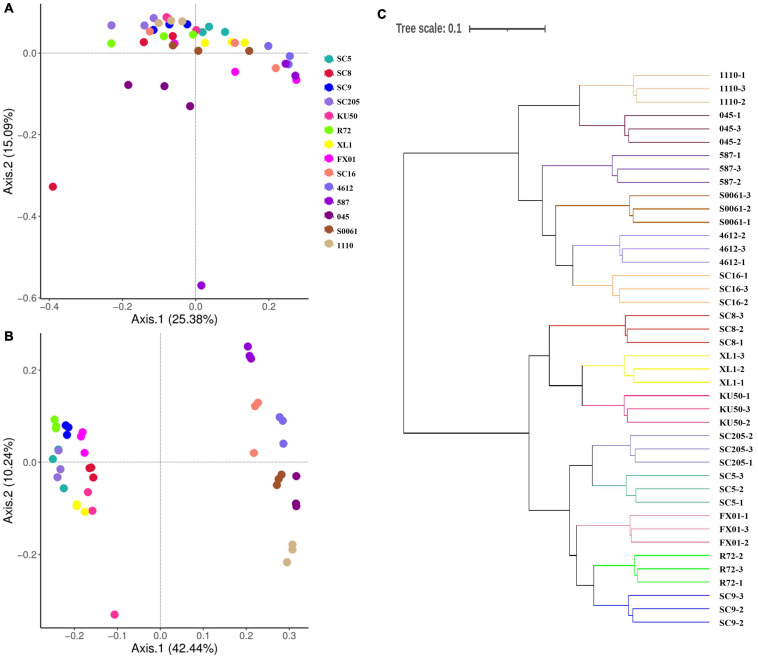
Principal coordinates analyses (PCoA) were performed based on **(A)** tuber and **(B)** rhizosphere soil bacterial OTU distributions using Bray-Curtis distance. **(C)** Hierarchical cluster analysis (HCA) was used to form a cluster tree of rhizosphere soil bacterial communities of different cassava genotypes.

A β-diversity analysis based on PCoA (Figures 5A,B) and HCA (Figure 5C) was performed to compare the bacterial compositions of the different samples. PCoA plots based on Bray-Curtis distances showed that bacterial communities in the tuberous roots were not clearly separated, and 40.47% (25.38 and 15.09%) of the overall variation could be explained. In contrast, the bacterial communities in the rhizosphere soil were clearly separated based on the cassava genotypes, which explained 52.68% (42.44 and 10.24%) of the overall variation (Figures 5A,B). Similar results were also obtained from the HCA tree. A cluster tree of all rhizosphere soil samples was constructed using HCA (Figure 5C). The bacterial communities in the rhizosphere soils of the different genotypes were clustered, and all branches were clustered based on the cassava genotypes. Thus, these results indicate that there is a correlation between the bacterial community in the rhizosphere of cassava and the genetic background of the cassava genotype.

### Bacterial Groups With Significant Differences Among Genotypes

In addition to characterizing their α- and β-diversities, another primary purpose of comparing the microbial communities was to identify specialized bacterial groups in the rhizosphere soils of each genotype. LEfSe can be used to analyze bacterial community data at any taxonomic level and to provide biological class explanations to establish statistical significance, biological consistency, and effect-size estimation of predicted biomarkers (Segata et al., 2011). We performed a statistical analysis of rhizosphere soil bacterial communities of the different cassava genotypes at the genus level, and a total of 323 distinct bacterial groups were identified using the default logarithmic LDA value of 2 (Supplementary Figure 2). The SC5 microbiome was characterized by the presence of *Bacillus* [LAD(log10) > 4.0]; SC8 was characterized by the presence of *Ktedonobacter* and *Gp2* [LAD(log10) > 4.0]; R72 was characterized by the presence of *Lactococcus* and *Burkholderia* [LAD(log10) > 4.0]; FX01 was characterized by the presence of *Thermosporothrix* and *Aciditerrimonas* [LAD(log10) > 4.0]; SC16 was characterized by the presence of *Sphingomonas* [LAD(log10) > 4.0]; 4612 was characterized by the presence of *Gaiella* and *Acidobacterium* [LAD(log10) > 4.0]; 587 was characterized by the presence of *Gp1*, *Subdivision3_genera_incertae_sedis* and *Gp6* [LAD(log10) > 4.0]; 045 was characterized by the presence of *Stella* and *Gp3* [LAD(log10) > 4.0]; S0061 was characterized by the presence of *Spartobacteria_genera_incertae_sedis* [LAD(log10) > 4.0]; and 1,110 was characterized by the presence of *Rhodoplanes* and *Spirillospora* [LAD(log10) > 4.0]. Interestingly, no bacterial genera from the rhizosphere of SC9, SC205, or KU50 had an LAD (log10) greater than 4.0.

### Influence of Tuber Metabolites on Microbial Communities

The α- and β-diversities of the tuber microbial communities were not significantly different among cassava genotypes, while the bacterial communities in the rhizosphere soils of KU50, SC205, and SC9 clustered into different groups. No bacterial genera had an LAD (log10) of greater than 4.0 for these three cassava genotypes, so they were selected for metabolome analysis.

A total of 78 compounds were detected in the tuber metabolites recovered from the tuberous roots of the three different cassava genotypes. The types of chemicals were the same across the three cassava genotypes. The identified compounds were categorized into nucleic acids, lipids, vitamins, cofactors, organic acids, peptides, and carbohydrates (Supplementary Figure 3A). Among them, the content of carbohydrates in the tuberous roots of SC9 was higher than that in the tuberous roots of the other genotypes; the content of peptides in the tuberous roots of KU50 was higher than that in the tuberous roots of the other genotypes; and the content of organic acids in the tuberous roots of SC205 was higher than that in the tuberous roots of the other genotypes. However, distinct differences in the abundances of certain compounds were detected (Supplementary Figure 3B), and the 19 most abundant metabolites in each of the three cassava genotypes were significantly different (*P* < 0.05) (Supplementary Table 6). PCA ordination showed that the tuber metabolite distributions of the three cassava genotypes were significantly separated from each other, indicating that the metabolite compositions and structures of the three cassava genotypes were quite different (Supplementary Figure 3C); the first two principal components of the PCA explained 73% (49.8 and 23.2%) of the total variation in the metabolites. To identify the metabolites that were notably different among the three cassava genotypes, OPLS-DA was performed on the metabolites of the three cassava genotypes. The analysis revealed that the metabolites with substantial differences among the three genotypes of cassava included sugars (5), sugar acids (4), sugar alcohols (2), organic acids (12), amino acids (3), amides/amines (4), and others (3) (Supplementary Figure 3D).

**FIGURE 6:**
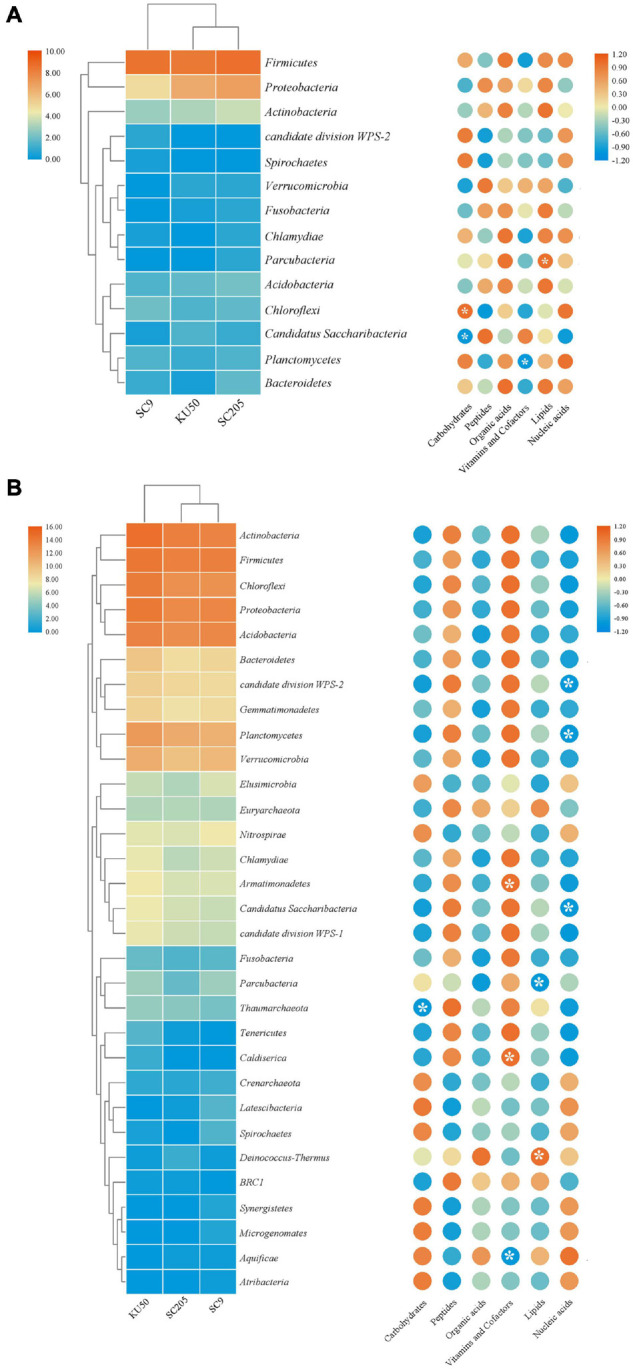
Relative abundance of bacterial phyla detected in **(A)** tuber and **(B)** rhizosphere soil of three cassava genotypes, and correlation analysis of bacterial community compositions and metabolite components. *Indicates significant differences (*P* < 0.05).

A correlation analysis between bacterial phyla and metabolites indicated that higher relative abundances of *Firmicutes*, *Proteobacteria*, and *Actinobacteria* were positively correlated with organic acids and lipids produced by the tuberous roots of the three cassava genotypes and negatively correlated with vitamins and cofactors (Figure 6A). Among the rhizosphere soils of the three cassava genotypes, higher relative abundances of most bacterial phyla were positively correlated with peptides, vitamins and cofactors and negatively correlated with carbohydrates, organic acids, lipids, and nucleic acids (Figure 6B). Further examination of the core genera, as defined by Bowen et al. (2017), indicated that tuber metabolites from the rhizosphere soil increased the relative abundances of 10 core bacterial genera, including *Lactococcus* and *Bacillus*, compared with those in tuberous roots. Interestingly, the identities and relative abundances of the core microbiome genera in the rhizosphere soil were both significantly different from those in the tuberous roots (Supplementary Table 4).

## Discussion

Plants and microbes interact in order to obtain nutrients to improve their growth and stress resistance, especially through root-microbe interactions (Edwards et al., 2015; Wu et al., 2020). Microbial communities have been shown to be impacted by the microhabitat (Jin et al., 2017), soil type (Bonito et al., 2014), and host genotype (Ofek-Lalzar et al., 2014; Zhang et al., 2019). At present, the microbial diversity of soils, roots, leaves, and aerial organs has been extensively investigated by high-throughput sequencing (Buée et al., 2009; Abdelfattah et al., 2016a,b, 2017; Liu et al., 2018; Yuan et al., 2018). To extend our knowledge of bacterial diversity as it relates to cassava genotypes, we used culture-independent high-throughput sequencing technology to investigate the diversity and community structure of bacteria present in the tuber endospheres and rhizospheres of fourteen cassava genotypes (SC5, SC8, SC9, SC205, KU50, R72, XL1, FX01, SC16, 4612, 587, 045, S0061, and 1110). Our results showed that the diversity of bacterial communities in the tuber endosphere and rhizosphere depends on the plant genotype and the tuber metabolites.

Previous reports have suggested that microbial density is generally higher in the rhizosphere than in the root and that bacterial diversity and richness gradually decrease from soils to epiphytes to endophytes (Bulgarelli et al., 2012, 2015; Lundberg et al., 2012; Edwards et al., 2015; Hacquard et al., 2015). In this study, the diversity and richness of bacteria in the rhizospheres of all cassava genotypes were higher than those in the tuber endospheres. Moreover, the cassava genotype did not significantly influence the endophytic bacterial community structure (Figure 4). Similar results have also been reported in previous studies: there were no significant differences in richness or diversity among the endophytes of different cassava genotypes (Li et al., 2020). Studies on *A. thaliana* have consistently suggested that root endosphere bacterial communities are strongly influenced by soil type and soil properties but that host genotype has a limited effect on the root microbiome (Bulgarelli et al., 2012; Lundberg et al., 2012; Thiergart et al., 2020). However, we detected a significant effect of cassava genotypes on the rhizosphere bacterial community structure (Figure 5), which is consistent with other previous findings (Miethling et al., 2000; Marschner et al., 2004; Garbeva et al., 2008; Bonito et al., 2014; Burns et al., 2015; Leff et al., 2018). For example, a significant effect of plant genotype on rhizosphere microbial communities was observed by comparing the rhizospheres of different experimental crops grown in soils of the same type (Liu et al., 2020). Some previous studies of the tree phyllosphere and maize rhizosphere separately showed that host genetics played an important role in shaping the bacterial microbiome (Laforest-Lapointe et al., 2016; Walters et al., 2018). Our results provide comprehensive empirical evidence for the selection of the microbial community by cassava and a theoretical framework for the coevolution between cassava and microbes; in this framework, cassava plants use exudates to recruit, filter, and enrich certain microbial taxa that have specific functions (Müller et al., 2016; Martin et al., 2017; Sasse et al., 2018), and competition among microbes for these resources drives their rapid evolutionary radiation and consequent divergence to reduce competition (Foster et al., 2017).

Generally, genetic based-interactions among genotypes are complex and have been recently gaining attention (Rasche et al., 2006; Xu et al., 2009; Aira et al., 2010; Ýnceoğlu et al., 2012; Cheng et al., 2020), and even minor genotype differences as between genetically modified and parental lines are believed to affect the microbial colonization of plant, particularly in vegetatively propagated crop. The seed stem-associated bacterial communities, independently of the genotypes and the soil type, is also a possible factor determining the specificity of the bacterial community in the tuber root system compartments. Nevertheless, the influence of genotype in our study is very evident.

In all cassava tuber samples, the dominant bacterial phyla were *Firmicutes*, *Proteobacteria*, and *Actinobacteria* (>1% of high-quality sequences) (Figure 1A). It has been previously reported that *Proteobacteria*, *Firmicutes*, and *Actinobacteria* are the dominant bacterial phyla in cassava (Li et al., 2020). Similar results based on both culture-dependent and culture-independent approaches have previously been reported for endophytes of other plants (Khan Chowdhury et al., 2017; Yang et al., 2017). In ginseng, *Proteobacteria* was found to have the highest abundance, followed by *Firmicutes* and *Actinobacteria* (Khan Chowdhury et al., 2017). Similarly, in peony, *Proteobacteria*, *Firmicutes*, and *Actinobacteria* have been reported to be the dominant bacterial phyla (Yang et al., 2017). In all cassava rhizosphere samples, the dominant bacterial phyla were *Actinobacteria*, *Proteobacteria*, *Acidobacteria*, *Firmicutes*, *Chloroflexi*, *Planctomycetes*, and *Verrucomicrobia* (>1% of high-quality sequences) (Figure 1A); these findings are in accordance with Sarr et al. (2017), who reported that the same soil bacterial communities were associated with cassava cultivation in Cameroon. *Actinobacteria* have also been shown to be enriched in exophytes of other plants, such as *Pinus pinaster* and maize-wheat (*Triticum aestivum*)/barley (*Hordeum vulgare*) rotation systems (Pérez-Izquierdo et al., 2019; Xiong et al., 2021); these bacteria are used as biocontrol agents to control soil- and seed-borne plant diseases (Priyadharsini and Dhanasekaran, 2015). These results suggest that rhizosphere microorganisms could play an important role in cassava cropping in tropical regions that experience various recurrent plant diseases.

The β-diversity analyses showed that bacterial communities in the rhizosphere soil varied across the different plant genotypes. PCoA indicated that the bacterial communities in the tuberous roots were not clearly separated by cassava genotype but that those in the rhizosphere soil were clearly separated by cassava genotype. These results were also supported by heatmap analyses at the genus level. Moreover, HCA demonstrated that the rhizosphere bacterial communities of the fourteen cassava genotypes were clustered based on the cassava genotypes (Figure 5C); these findings support the view that the cassava genotype influences the bacterial rhizosphere community. Similarly, Schlemper et al. (2017) reported that the community structures of the rhizosphere microbiome were significantly different among seven different sorghum cultivars. However, further studies are needed to confirm this hypothesis and to confirm the effects of genetic diversity on the compositions of root-associated bacterial communities.

We noted a few genera that were consistently enriched in the cassava tuberous roots, including *Lactococcus*, *Bacillus*, *Oceanobacillus*, and *Carnobacterium* (Figure 2A). *Lactococcus* and *Bacillus* improve plant resistance to diseases such as bacterial blight and root rot in cassava and wilt disease in cucumber (Xu et al., 2014; Freitas et al., 2019; Zhang et al., 2021). In addition, *Pseudomonas* was also a dominant genus in the tuber endosphere of 045. *Pseudomonas* species have been used to alleviate heavy metal toxicity and the negative effects of saline sodic field growth on wheat (Hassan et al., 2017). Eight genera (*Gp1*, *Gaiella*, *Bacillus*, *Lactococcus*, *Gp3*, *Ktedonobacter*, *Rhodoplanes*, and *Spirillospora*) were highly abundant in all rhizosphere samples (Figure 2B). This result is in accordance with Bao et al. (2019), who reported that the dominant bacteria in a paddy soil included *Bacillus*, *Acidobacteria/Gp1*, *Acidobacteria/Gp3*, and *Ktedonobacter*. Wu et al. (2021) found that the enrichment of beneficial bacteria, mainly *Gaiella*, contributed to the ability of ramie to tolerate poor soils. Our results showed that the cultivation of different cassava genotypes recruited different unique and core microbes to the cassava tubers and rhizospheres and that the microbes were significantly different in identity and relative abundance (Figure 3). This result is mainly attributed to the process by which cassava plants recruit different microbes, i.e., the release of a wide variety of exudates from tubers (Garbeva et al., 2004; Raaijmakers et al., 2009). LEfSe analysis identified specialized bacterial groups exclusively in the rhizosphere soil (Supplementary Figure 2), which suggests that these groups may play critical roles in maintaining the structure and function of rhizospheric soil bacterial communities. For example, *Burkholderia*, found in the R72 rhizosphere, was more abundant in a pineapple-banana crop rotation soil than in a banana monoculture soil, has the capacity to suppress Fusarium wilt of banana (Wang et al., 2015). Other reports who have demonstrated that bacteria from the *Burkholderia* genera possess a potential biocontrol ability through the production of varying compounds that inhibit plant pathogens (Mendes et al., 2011; Raaijmakers and Mazzola, 2012; Tenorio-Salgado et al., 2013). *Spartobacteria_genera*, found in the S0061 rhizosphere, was stimulated potentially to suppress the Fusarium wilt disease by sustainable biofertilizer application (Shen et al., 2015). Furthermore, we also found that most core microbes in the tubers and rhizosphere had plant growth-promoting potential. These core genera are known to produce various antibiotics, including bacillibactin and lipopeptides (produced by *Bacillus*) (Li et al., 2014; Liu et al., 2017), 2, 4-diac-etylphlor-oglucinol and phenazines (produced by *Pseudomonas*) (Mazurier et al., 2009; Hu et al., 2017), fusaricidin (produced by *Paenibacillus*) (Finch et al., 2018; Li and Chen, 2019), and thiopeptide and ectoine (produced by *Streptomyces*) (Cha et al., 2016). Overall, identifying these core and unique microbiomes is important for understanding the responsive microbial components associated with different plant genotypes.

In this study, the bacterial communities in the rhizosphere soils conditioned by the tuber metabolomes collected from three typical cassava genotypes, KU50, SC205, and SC9, were significantly different from each other taxonomically; in contrast, the bacterial communities in the tuberous roots were not significantly different. The correlation analysis between bacterial phyla and the produced exudates revealed that these differences could be linked to the exudation of certain tuber metabolites (Figure 6). These results thus support the notion that specific compounds within changing plant exudate profiles may drive soil microbial dynamics (Badri et al., 2013). Although the potential to mobilize soil nutrients is clearly already present in the soil microbiome, tuber exudates increase the functional potential of soil microbial communities. In addition, exudate concentrations can play a major role in shaping the abundances of microbial functional genes, which may be beneficial to plants (Badri et al., 2013). Moreover, the contents of measured metabolites in the three genotypes of cassava and their biological roles were significantly different; the metabolites included sugars, sugar acids, sugar alcohols, organic acids, amino acids, amides/amines, and others (Supplementary Figure 3). These differences could reflect a level of microbial community functional redundancy that was stimulated by the metabolomes. In a study of the rhizosphere microbiome of *A. thaliana*, Chaparro et al. (2013) suggested that plants exude sugars that are used by a wide variety of microorganisms as well as more specific exudates, such as phenolic compounds, that may be intended to attract more specific microbes. Amino acids, as specific chemoattractants for microorganisms, promote the chemotaxis of soil microbes to the rhizosphere (Barbour et al., 1991). Organic acids play a crucial role in nutrient acquisition (P, Fe, and Mn) by plants growing in nutrient-poor soils (Dakora and Phillips, 2002). Our result is in accordance with the finding that specific exudates of different *Sorghum bicolor* genotypes may influence the rhizosphere microbial community composition (Funnell-Harris et al., 2008). The available mineral nutrients in soils are not sufficient to meet the requirements of plants for optimal growth; thus, plants have evolved systems to recruit symbiotic microbial partners that increase the availability of nutrients (Tinker, 1984; Landeweert et al., 2001; Gyaneshwar et al., 2002; Adesemoye and Kloepper, 2009). Many studies of root exudate-mediated microbial defenses have developed robust models of coevolution between plants and soil microbes; for example, in the “cry for help” and “legacy effects” models (Weller et al., 2002; Yuan et al., 2018), plants select for microbial communities that help to suppress plant pathogens. The exudates released by different genotypes of the same plant species can vary, which affects the microbial community composition of the rhizosphere (Micallef et al., 2009; Inceoğlu et al., 2010).

## Conclusion

The different cassava genotypes did not affect the richness or diversity of the endophytic bacterial community, but they affected the richness and diversity of the exophytic bacterial community. Furthermore, the cassava genotype shaped the endophytic and exophytic community structures and affected the relative abundances of core bacterial genera. The bacterial community structures varied between the tuber endosphere and the rhizosphere across cassava genotypes. The more dominant bacterial phyla associated with tubers and the rhizosphere were *Firmicutes* and *Actinobacteria*, respectively. Moreover, the majority of genera were associated with the tuberous roots and rhizosphere soils of specific cassava genotypes; this may have been due to the exudation of certain metabolites from the cassava tubers. These results suggest that plant genotypes affect the community composition of endophytic bacteria and may affect the community composition of exophytic bacteria through the exudation of metabolites.

## Data Availability Statement

The raw sequences were deposited in the NCBI Sequence Read Archive under BioProject accession number PRJNA750582.

## Author Contributions

YC and KL conceived and designed the experiments. JH, YG, RZ, YZ, XN, and XC performed the experiments and data analyses. JH drafted the manuscript. JH, YG, YC, and KL revised the manuscript. All authors have read and approved the submitted version.

## Conflict of Interest

The authors declare that the research was conducted in the absence of any commercial or financial relationships that could be construed as a potential conflict of interest.

## Publisher’s Note

All claims expressed in this article are solely those of the authors and do not necessarily represent those of their affiliated organizations, or those of the publisher, the editors and the reviewers. Any product that may be evaluated in this article, or claim that may be made by its manufacturer, is not guaranteed or endorsed by the publisher.

## Footnotes

1
http://gmd.mpimp-golm.mpg.de/
