# Supplementary material for: Diversity of the Bacterial Microbiome Associated With the Endosphere and Rhizosphere of Different Cassava (Manihot esculenta Crantz) Genotypes
Source: Front Microbiol. 2021 Sep 30;12:729022. doi: 10.3389/fmicb.2021.729022 (PMC8515189; doi:10.3389/fmicb.2021.729022)

FIGURE S1 | The rarefaction curves of tuber and rhizosphere samples of different cassava genotypes.

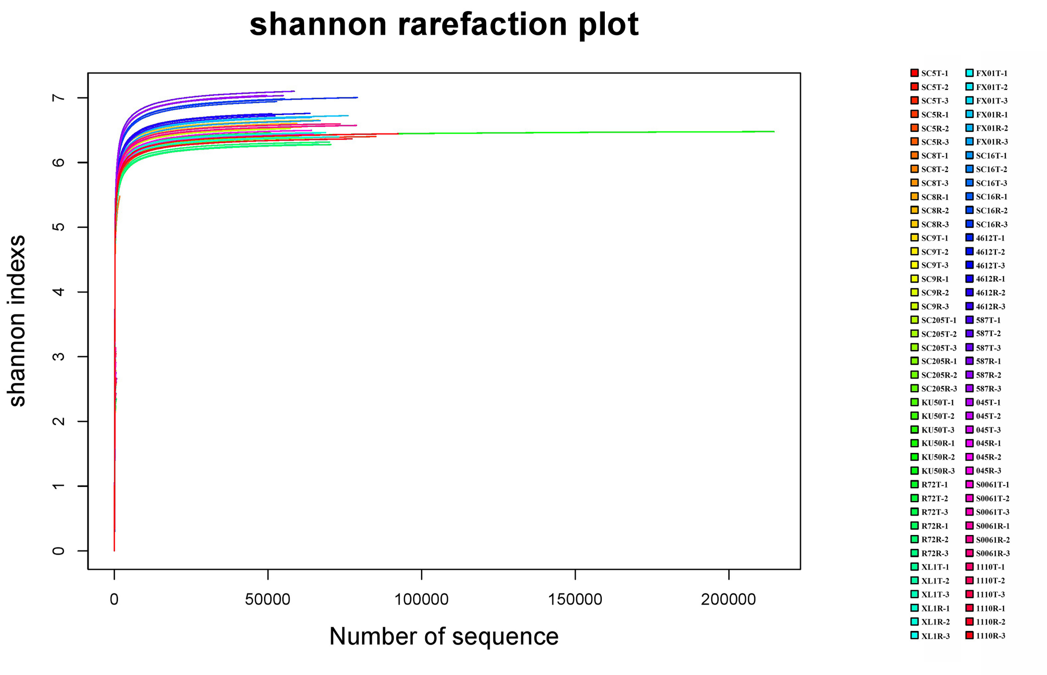

FIGURE S2 | Bar diagram of the linear discriminant analysis (LDA) distribution (LDA score threshold: ≥2) with rhizosphere soil bacterial communities.

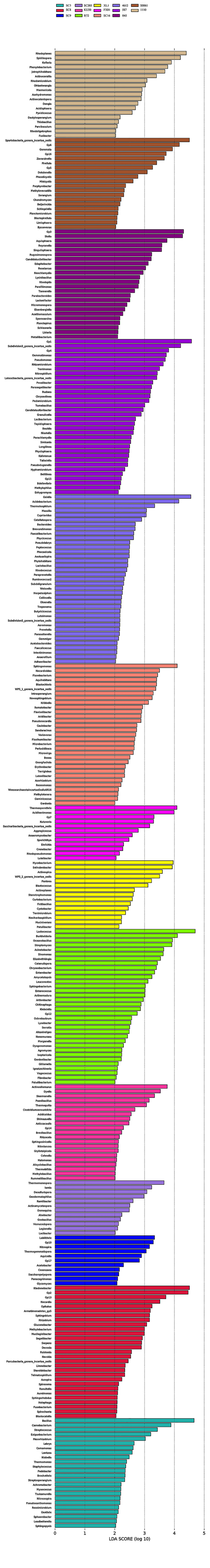

FIGURE S3 | (A) Unsupervised PCA diagrams of three cassava cultivars. Stacked column chart of the percentage of metabolites playing a biological role (B) and the Top 20 metabolite percentages (C) of three cassava cultivars. (D) The importance map of OPLS-DA metabolites of three cassava cultivars. The metabolites in the yellow area were significantly different between groups ($P < 0.05$, VIP > 1).

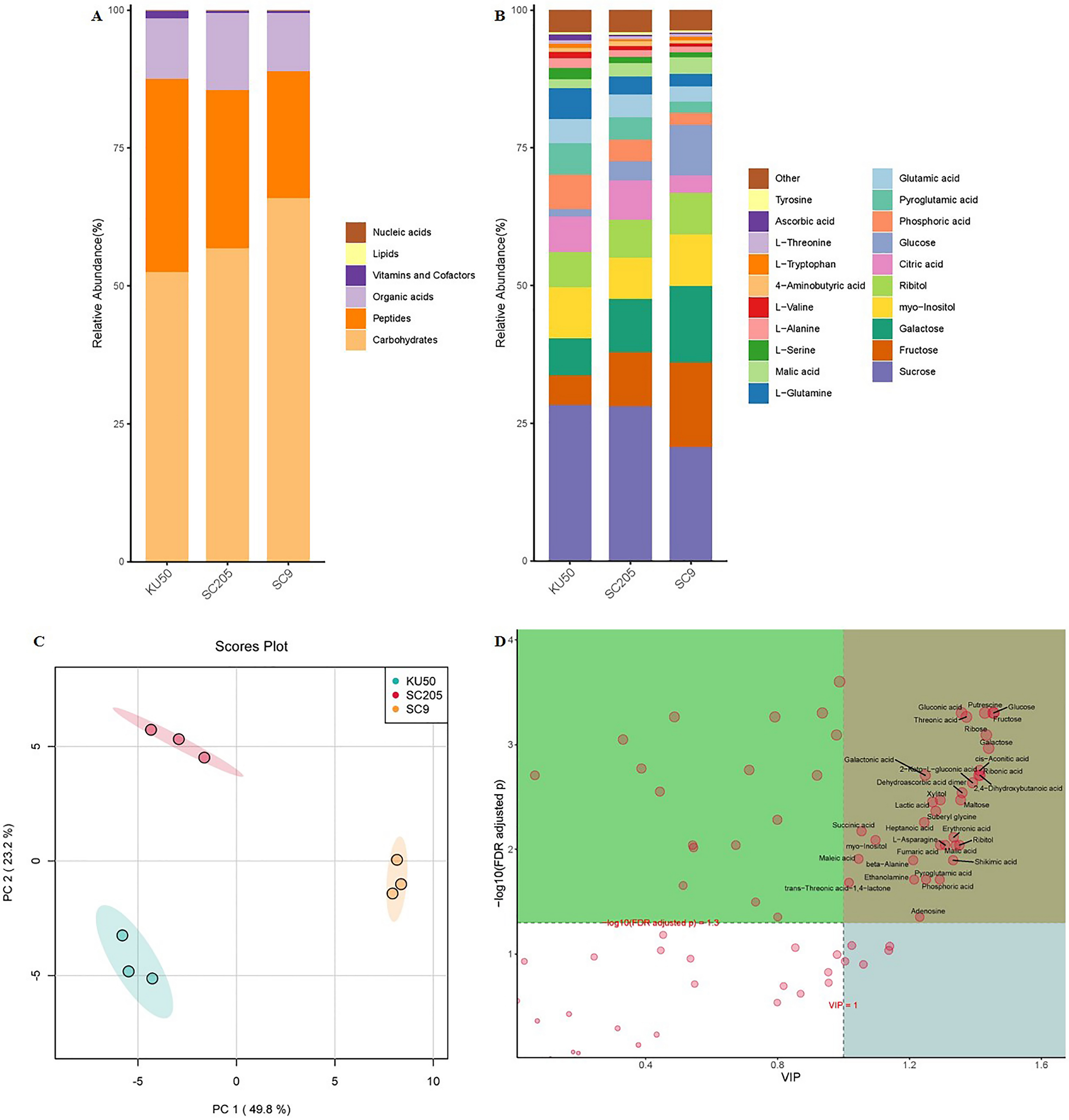

Supplement: Supplementary file 2 [file Presentation_1.pdf]
